# Activation of Astrocytes in the Persistence of Post-hypoxic Respiratory Augmentation

**DOI:** 10.3389/fphys.2021.757731

**Published:** 2021-10-08

**Authors:** Isato Fukushi, Kotaro Takeda, Mieczyslaw Pokorski, Yosuke Kono, Masashi Yoshizawa, Yohei Hasebe, Akito Nakao, Yasuo Mori, Hiroshi Onimaru, Yasumasa Okada

**Affiliations:** ^1^Faculty of Health Sciences, Uekusa Gakuen University, Chiba, Japan; ^2^Clinical Research Center, Murayama Medical Center, Musashimurayama, Japan; ^3^Faculty of Rehabilitation, School of Healthcare, Fujita Health University, Toyoake, Japan; ^4^Institute of Health Sciences, University of Opole, Opole, Poland; ^5^Faculty of Health Sciences, The Jan Dlugosz University in Czestochowa, Czestochowa, Poland; ^6^Department of Pediatrics, Faculty of Medicine, University of Yamanashi, Yamanashi, Japan; ^7^Laboratory of Molecular Biology, Department of Synthetic Chemistry and Biological Chemistry, Graduate School of Engineering, Kyoto University, Kyoto, Japan; ^8^Department of Physiology, Showa University School of Medicine, Tokyo, Japan

**Keywords:** astrocyte, hypoxia, post-hypoxic respiratory augmentation, plasticity, short-term potentiation, respiratory control, arundic acid, TRPA1

## Abstract

Acute hypoxia increases ventilation. After cessation of hypoxia loading, ventilation decreases but remains above the pre-exposure baseline level for a time. However, the mechanism of this post-hypoxic persistent respiratory augmentation (PHRA), which is a short-term potentiation of breathing, has not been elucidated. We aimed to test the hypothesis that astrocytes are involved in PHRA. To this end, we investigated hypoxic ventilatory responses by whole-body plethysmography in unanesthetized adult mice. The animals breathed room air, hypoxic gas mixture (7% O_2_, 93% N_2_) for 2min, and again room air for 10min before and after i.p. administration of low (100mg/kg) and high (300mg/kg) doses of arundic acid (AA), an astrocyte inhibitor. AA suppressed PHRA, with the high dose decreasing ventilation below the pre-hypoxic level. Further, we investigated the role of the astrocytic TRPA1 channel, a putative ventilatory hypoxia sensor, in PHRA using astrocyte-specific *Trpa1* knockout (as*Trpa1*^−/−^) and floxed *Trpa1* (*Trpa1*^f/f^) mice. In both *Trpa1*^f/f^ and as*Trpa1*^−/−^ mice, PHRA was noticeable, indicating that the astrocyte TRPA1 channel was not directly involved in PHRA. Taken together, these results indicate that astrocytes mediate the PHRA by mechanisms other than TRPA1 channels that are engaged in hypoxia sensing.

## Introduction

Acute hypoxia increases ventilation. After brief hypoxic exposure, a switchback to room air is accompanied by a ventilatory fall-off in the recovery phase, but ventilation remains above the pre-hypoxic baseline for a time. Post-hypoxic persistent respiratory augmentation (PHRA) is a form of neural plasticity, which is defined as a change in the neural control system based on the memory-like experience (Mitchell and Johnson, 2003). The poststimulus overshoot in ventilatory activity may even go above the stimulus level as is evident in the acute hypoxic ventilatory response (HVR) to static exercise, with the mechanism ascribed to the interaction with the cardiovascular brain control or rapid release of the volitional hypothalamic control over sustained muscle tension (Pokorski et al., 1990). Neural plasticity is essential for stabilizing respiratory control, but the underlying mechanisms are not yet well known (Eldridge and Millhorn, 1986; Dahan et al., 1995; Eldridge, 1996; Powell et al., 1998).

There are plastic interactions in relay circuits of hypoxic stimulus between peripheral chemoreceptors, among which carotid body chemoreceptors are most engaged in creating the HVR, and brain respiratory control pathways (Pamenter and Powell, 2016). The multipronged complexity of PHRA is highlighted by increased carotid chemoreceptor sensitivity due to the withdrawal of the central efferent activity component running down the sinus nerve to the carotid body (Lahiri et al., 1983). That feature has been unraveled in adaptive plasticity to chronic hypoxia but is plausibly also present in repeat acute hypoxic episodes characteristic of sleep apnea syndrome, the disease that distinctly affects brain function and increases chemoreflex sensitivity (Prabhakar, 2016).

Limited understanding of peripheral and central underliers of respiratory plasticity spurred novel lines of research, one of which is the role of transient receptor potential ankyrin 1 (TRPA1) channel. These channels participate in shaping the acute HVR (Pokorski et al., 2014). However, the channels have never been verified in carotid chemoreceptor cells and their effects on the HVR are mediated by mechanisms other than the carotid body (Pokorski et al., 2014). It has been shown that TRPA1 is localized in the chemosensitive parafacial respiratory group (pFRG/RTN) astrocytes in which hypoxia-induced TRPA1 activation facilitates exocytosis of ATP-containing vesicles (Uchiyama et al., 2020). On the basis of these findings, TRPA1 channels in astrocytes have been proposed as an oxygen sensor for respiratory control (Uchiyama et al., 2020). The proposition is in line with studies that show the role of astrocytes in brain synaptic plasticity (De Pittà et al., 2016; Schiera et al., 2020). Astrocytes are also influential for various aspects of respiratory control, including rhythm generation (Okada et al., 2012; Sheikhbahaei et al., 2018) and hypoxic and hypercapnic ventilatory responses (Gourine et al., 2010; Funk et al., 2015; Pokorski et al., 2016; Beltrán-Castillo et al., 2017; Gourine and Funk, 2017; Funk and Gourine, 2018; Sheikhbahaei et al., 2018; Guyenet et al., 2019). It has been reported that astrocytes can detect hypoxia (Tadmouri et al., 2014; Angelova et al., 2015; Fukushi et al., 2016; Onimaru et al., 2021). Therefore, we aimed to test the hypothesis that astrocytes are involved in PHRA and define the role of astrocytes, notably through TRPA1 channels, in the PHRA phenomenon. We used arundic acid (AA) as a pharmacological tool to inhibit astrocytic function in wild-type mice. We also used astrocyte *Trpa1* knockout mice to investigate the role of astrocytic TRPA1 channels in PHRA. We found that the presence of active astrocytes is indispensable for the expression of PHRA, but their action is mediated by mechanisms other than TRPA1 channels.

## Materials and Methods

### Animal Welfare

All animal experiments were performed with the approval of the Ethics Committee for Animal Experiments of the Murayama Medical Center in Tokyo and complied with the Guidelines for Care and Use of Laboratory Animals released by the National Research Council of the National Academies (8th edition, revised 2011) and with the Guiding Principles for Care and Use of Animals of the Physiological Society of Japan. A total of 34 mice (including the mice in experiments for Supplementary Figures 1, 2) were used in the experiments. All efforts were made to minimize the number of animals used.

### Experiments With Arundic Acid

We used unanesthetized adult male C57BL/6 mice aged 24.0±3.0weeks (mean±SE). It should be the same weeks, weighing 29.6±0.7g (*n*=9). The respiratory flow was measured noninvasively using an “open flow” whole-body plethysmograph (PLY 310, EMMS, Bordon, United Kingdom) consisting of recording (volume of 530ml) and reference chambers as previously described (Oyamada et al., 2008; Pokorski et al., 2014; Fukushi et al., 2016, 2020). Briefly, the chambers were placed inside a transparent acrylic box (size 20×20×20cm). Each mouse was placed in the pre-calibrated recording chamber. The chamber temperature was maintained at 25°C throughout. The air in the recording chamber was suctioned with a constant flow generator (MV-6005VP, E.M.P-Japan, Tokyo, Japan), with a flow rate of 250ml/min. To calculate the airflow, the pressure difference between the recording and reference chambers was measured with a differential pressure transducer (TPF100, EMMS) connected to an amplifier (AIU060, Information & Display Systems, Bordon, United Kingdom) and was bandpass filtered at 0.1–20Hz. We calculated tidal volume (V_T_; μl/g b.w.) for each breath by integrating the airflow whose changes are proportional to those in the chamber pressure (Lundblad et al., 2002). We counted the number of breaths and obtained respiratory rate (RR; breaths/min). Minute ventilation (V_E_; ml/g/min) was calculated as V_T_×RR for each minute. The V_E_ during hypoxia was calculated as a 2-min average and during the recovery phase as an average of the first 5min (Recovery 1) and second 5min (Recovery 2). The O_2_ concentration in the chamber was monitored with an O_2_ analyzer incorporating a polarographic sensor (Respina IH 26, San-ei, Tokyo, Japan) and was adjusted by controlling the mixing of N_2_ and air blown into the acrylic box. The pressure and O_2_ concentration data were simultaneously digitized at a 400Hz sampling rate with an A/D converter (PowerLab4/26) and stored in a PC with LabChart7 software. The signal processing was performed using MATLAB 2020a (MathWorks, Natick, MA).

To evaluate the HVR, mice breathed room air, then a hypoxic gas mixture (7% O_2_, 93% N_2_ for 2min), and room air again before and after i.p. administration of AA. The experimental protocol consisted of three repeats of hypoxic challenges. First, dimethyl sulfoxide (DMSO), a vehicle for AA diluted in saline, was injected and the mouse was placed into the chamber to acclimatize in room air for 60min. Then, after recording normoxic baseline data for 1min, N_2_ gas was blown into the acrylic box. The chamber O_2_ concentration rapidly declined to 7%, which was maintained for 2min and followed by a switchback to room air. The measurement for the recovery continued for 10min. This protocol was repeated after injections of two doses of AA solubilized in a mixture of DMSO and saline (1:4:5 *v*/*v*) at 30-min intervals. Thus, injections were made in the following sequence (1) vehicle – 0.45ml/kg DMSO, (2) AA – 100mg/kg, and (3) AA – 200mg/kg (cumulative AA dose of 300mg/kg). Although DMSO alone can affect the brain function when the dose is high, a total dose of DMSO used in the present experiment did not exceed 2.0g/kg, which is much below the 3.5g/kg, a dose that starts affecting respiration (Takeda et al., 2016). The total volume of saline used in the experiment was 2.24ml/kg, which is much below the 10ml/kg reported to affect respiration in mice (Receno et al., 2018). The dosing of AA was chosen according to previous studies using this agent in *in-vivo* rodents (Higashino et al., 2009; Fukushi et al., 2016, 2020). Any apparent movement and sniffing artifacts interfering with breathing patterns were discarded off-line from the recording traces during the final data elaboration. The mean values of V_E_ were submitted to a two-factor within-subject analysis of variance (ANOVA), with three pharmacological conditions: DMSO vehicle and the two doses of AA, and four air phases (Baseline room air, Hypoxia, Recovery 1, and Recovery 2). The same statistical tests were performed for RR and V_T_ as for V_E_. A Greenhouse–Geisser adjustment was used to correct for violations of sphericity whenever necessary. Then, to quantitatively evaluate the magnitude of PHRA, we calculated the difference in V_E_ between the post-hypoxic recovery and pre-hypoxic baseline levels. This difference was divided by the difference in V_E_ between the hypoxic loading and pre-hypoxic levels to normalize for the degree of hypoxic ventilatory augmentation. The calculation provided the parameter ΔV_ERecovery_/ΔV_EHypoxia_ to compare the PHRA magnitude among three drug conditions (without AA and with low and high doses of AA) in the post-hypoxic Recovery 1 and Recovery 2 phases. Statistical differences were assessed with a paired t-test. Bonferroni correction was performed for the multiple comparisons.

### Experiments Using Astrocyte-Specific *Trpa1* Knockout Mice

We examined the role of astrocyte TRPA1 channels in HVR and PHRA using astrocyte-specific *Trpa1* knockout mice (as*Trpa1*^−/−^). To generate the as*Trpa1*^−/−^, two lines of mice were crossed: a transgenic mouse GFAP-Cre (mGFAP-Cre) and a recombinant *Trpa1* floxed (*Trpa1*^f/f^) mouse (Gregorian et al., 2009; Zappia et al., 2017; Uchiyama et al., 2020). We conducted 7% hypoxia loading experiments in as*Trpa1*^−/−^ mice (seven males and five females, aged 21.8±0.4weeks, weighing 25.8±1.1g) and *Trpa1*^f/f^ mice (two males and four females, aged 22.7±1.3weeks, weighing 24.2±0.9g) according to the same protocol and measurement methods as outlined above for the AA experiments. The mean values of V_E_, V_T_, and RR were submitted to two-way ANOVA with two TRPA1 conditions (as*Trpa1*^−/−^ and *Trpa1*^f/f^) as between-factor and with four air phases (Baseline room air, Hypoxia, Recovery 1, and Recovery 2) as within-factor. A Greenhouse–Geisser adjustment was used to correct for violations of sphericity. We calculated the ΔV_ERecovery_/ΔV_EHypoxia_ to compare the PHRA magnitude between the two TRPA1 conditions (as*Trpa1*^−/−^ and *Trpa1*^f/f^) in the post-hypoxic Recovery 1 and Recovery 2 phases using the Welch test. The Bonferroni correction was used for multiple comparisons in *post hoc* tests. A *p*<0.05 defined statistically significant differences. The analysis was performed using SPSS 24.0 (IBM, Armonk, NY).

## Results

### Effects of Arundic Acid on HVRs

**Figure 1:**
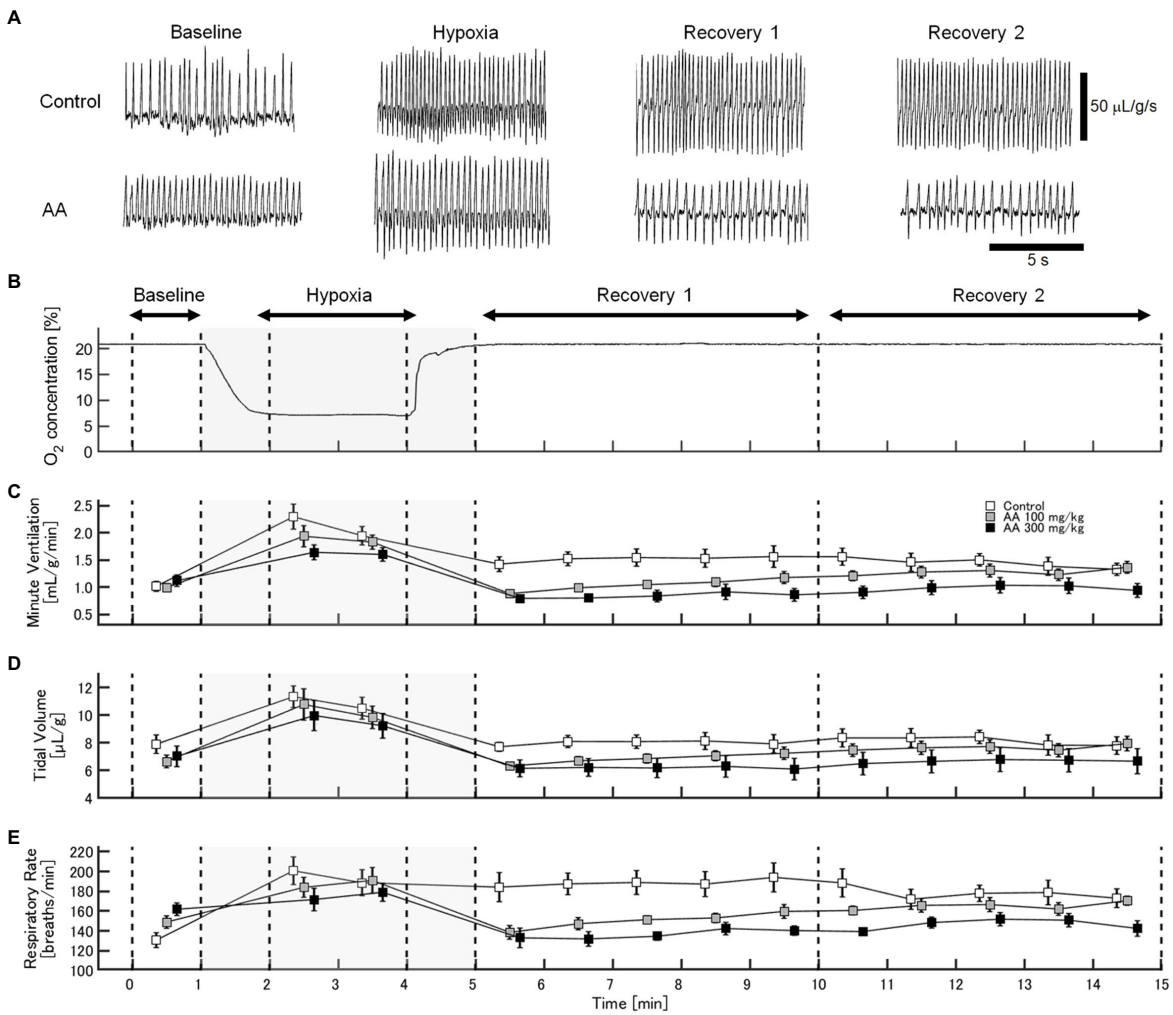
Effects of arundic acid (AA) on hypoxic ventilatory responses (HVRs) in mice by whole-body plethysmography. **(A)** Representative recordings of respiratory flow (inspiration upward) in mice without and with the higher dose of AA in room air (baseline), 7% hypoxia, Recovery 1 (first 5min), and Recovery 2 (second 5min). AA did not affect minute ventilation (V_E_) at pre-hypoxic baseline but tended to suppress the acute hypoxic hyperventilation. After the lower AA dose, V_E_ immediately returned to the pre-hypoxic baseline in Recovery 1 but rebounded in Recovery 2. After the higher AA dose, V_E_ decreased below the pre-hypoxic baseline in Recovery 1 and tended to revert to the baseline level in Recovery 2 failing to reach it. **(B)** Time-profile of chamber oxygen changes. **(C–E)** Time-series data for minute ventilation, tidal volume, and respiratory rate (RR), respectively, in the control (no AA) and low- and high-AA dose conditions.

**Figure 2:**
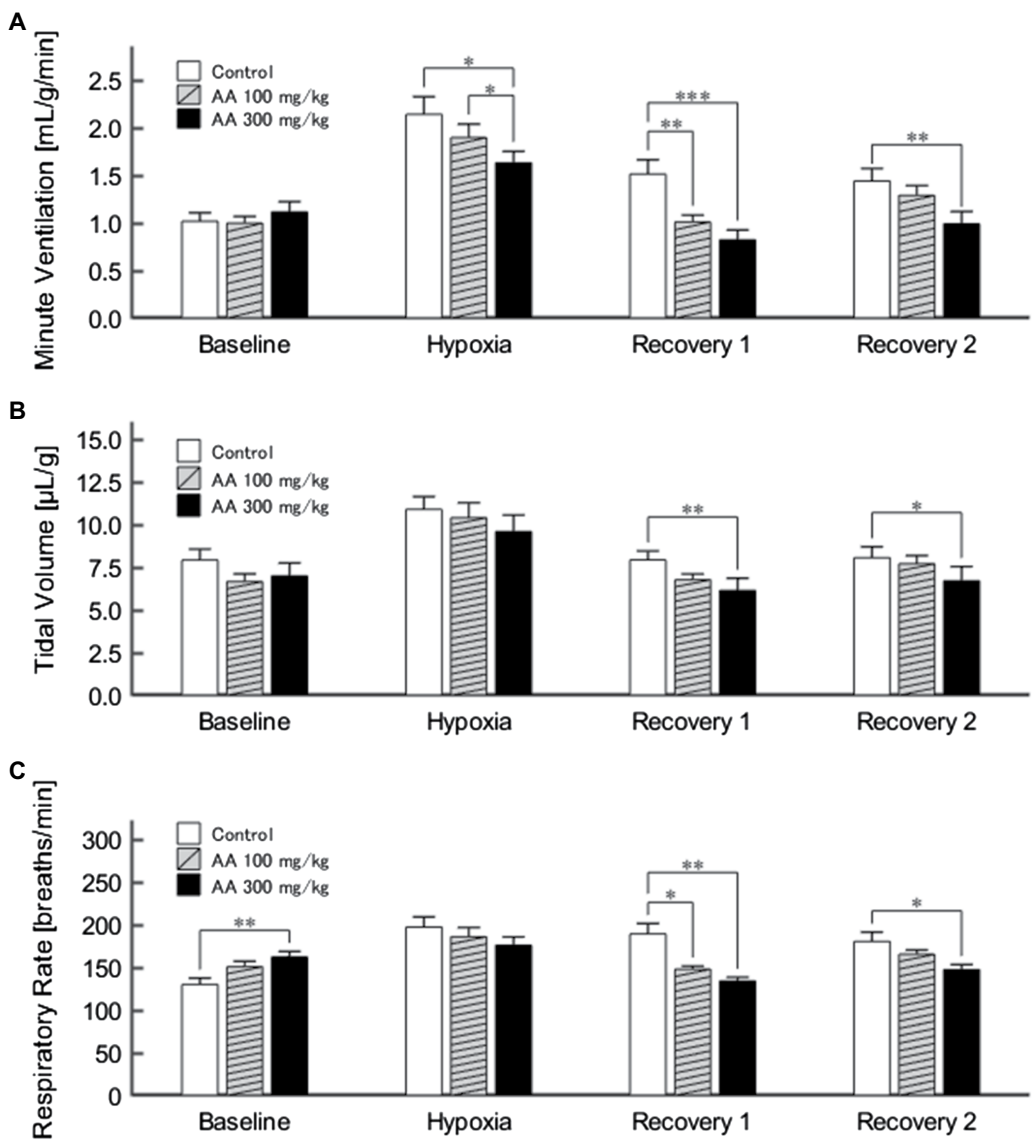
**(A)** Minute ventilation (V_E_) profiles (*n*=9) in the control (no AA) and low- and high-AA dose conditions across the successive ventilatory phases. V_E_ differed significantly in the following pairwise comparisons: Control vs. AA 100 and AA 100 vs. AA 300 in hypoxia (both *p*<0.05); Control vs. AA 100 (*p*<0.01) and Control vs. AA 300 (*p*<0.001) in Recovery 1; and Control vs. AA 300 (*p*<0.01) in Recovery 2. **(B)** The time-course of tidal volume (V_T_). There were main effects on V_T_ of the AA condition (*F*_2,16_=6.596, *p*<0.01) and oxygen concentration (*F*_3,24_=89.579, *ε*_GG_=0.424, *p*<0.001), but no interaction between the two (*F*_6,48_=1.205, *ε*_GG_=0.522, *p*=0.329). V_T_ differed significantly in the following comparisons: Control vs. AA 300 in Recovery 1 and Control vs. AA 300 in Recovery 2 (both *p*<0.01). **(C)** Time-course of RR. There was a significant interaction between control (no AA) and two AA conditions×HVR phases (*F*_6,42_=12.208, *p*<0.001). RR differed significantly in the following comparisons: Control vs. AA 300 at baseline (*p*<0.01); Control vs. AA 100 (*p*<0.05) and Control vs. AA 300 (*p*<0.01) in Recovery 1; and Control vs. AA 300 (*p*<0.05) in Recovery 2. ^*^p<0.05, ^**^p<0.01, and ^***^p<0.001, Bonferroni corrected.

**Figure 3:**
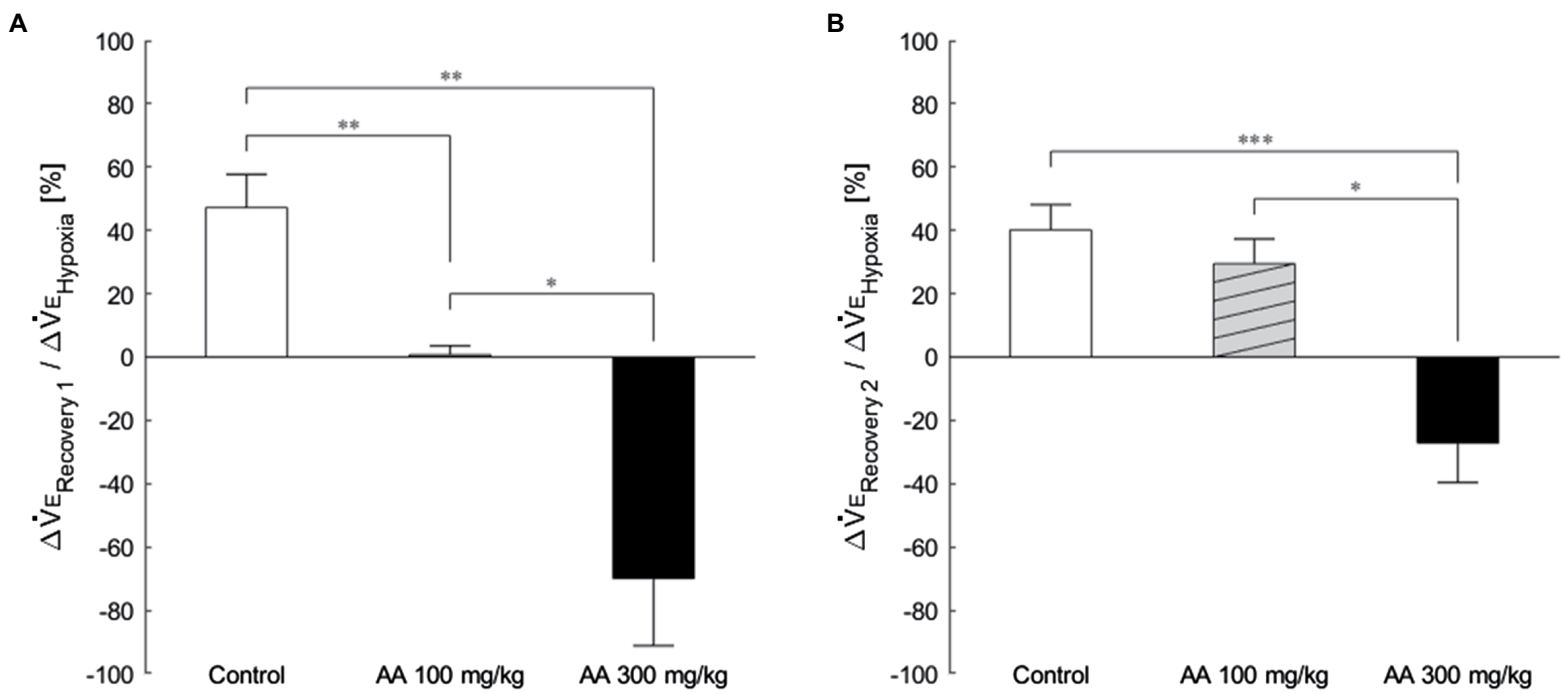
The magnitude of effects of AA on post-hypoxic persistent respiratory augmentation (PHRA) evaluated according to the formula ΔV_ERecovery_/ΔV_EHypoxia_ (see Materials and Methods for details). **(A)** Recovery 1 – PHRA differed between control (no AA) vs. AA 100, control vs. AA 300, and AA 100 vs. AA 300 conditions. **(B)** Recovery 2 – PHRA differed between control (no AA) vs. AA 300. Data are means±SE. ^*^p<0.05, ^**^p<0.01, and ^***^p<0.001, Bonferroni corrected.

The exemplary recordings of V_E_ profiles in the two AA conditions vs. the control condition with no AA across the baseline room air, hypoxia, and Recovery 1 and 2 phases are shown in Figure 1. There was a significant interaction between pharmacological conditions×ventilatory phases [*F*(6, 42)=8.08, *p*<0.001]. On average, AA failed to affect V_E_, despite some increases in RR after the higher dose of AA in room air. While V_E_ increased during hypoxia on the background of AA, there were differences in the post-hypoxia recovery course. In the control condition, V_E_ decreased from the hypoxic hyperventilation level but remained higher than the pre-hypoxic baseline level in both recovery phases. In the low-dose AA condition, V_E_ immediately returned to the pre-hypoxic baseline level during Recovery 1 but increased again above it during Recovery 2. In the high-dose AA condition, V_E_ decreased significantly below the pre-hypoxic baseline level during Recovery 1 and then tended to revert to the baseline level in Recovery 2 failing to reach it. The time courses of V_E_, V_T_, and RR as outlined in the example shown above are summarized in Figure 2. Figure 3A shows that the ΔV_ERecovery_/ΔV_EHypoxia_, assessing the PHRA magnitude, was significantly smaller in Recovery 1 between control (no AA) and 100mg/kg AA (*p*<0.01) or 300mg/kg AA (*p*<0.001), and between 100 and 300mg/kg AA (*p*<0.05). The differences between control (no AA) and 300mg/kg AA and between 100 and 300mg/kg AA distinctly persisted in Recovery 2 (Figure 3B). Thus, blockade of astrocyte activation significantly attenuated PHRA; the effect was greatly potentiated at the higher AA dose.

### HVRs in as*Trpa1*^−/−^ Mice

**Figure 4:**
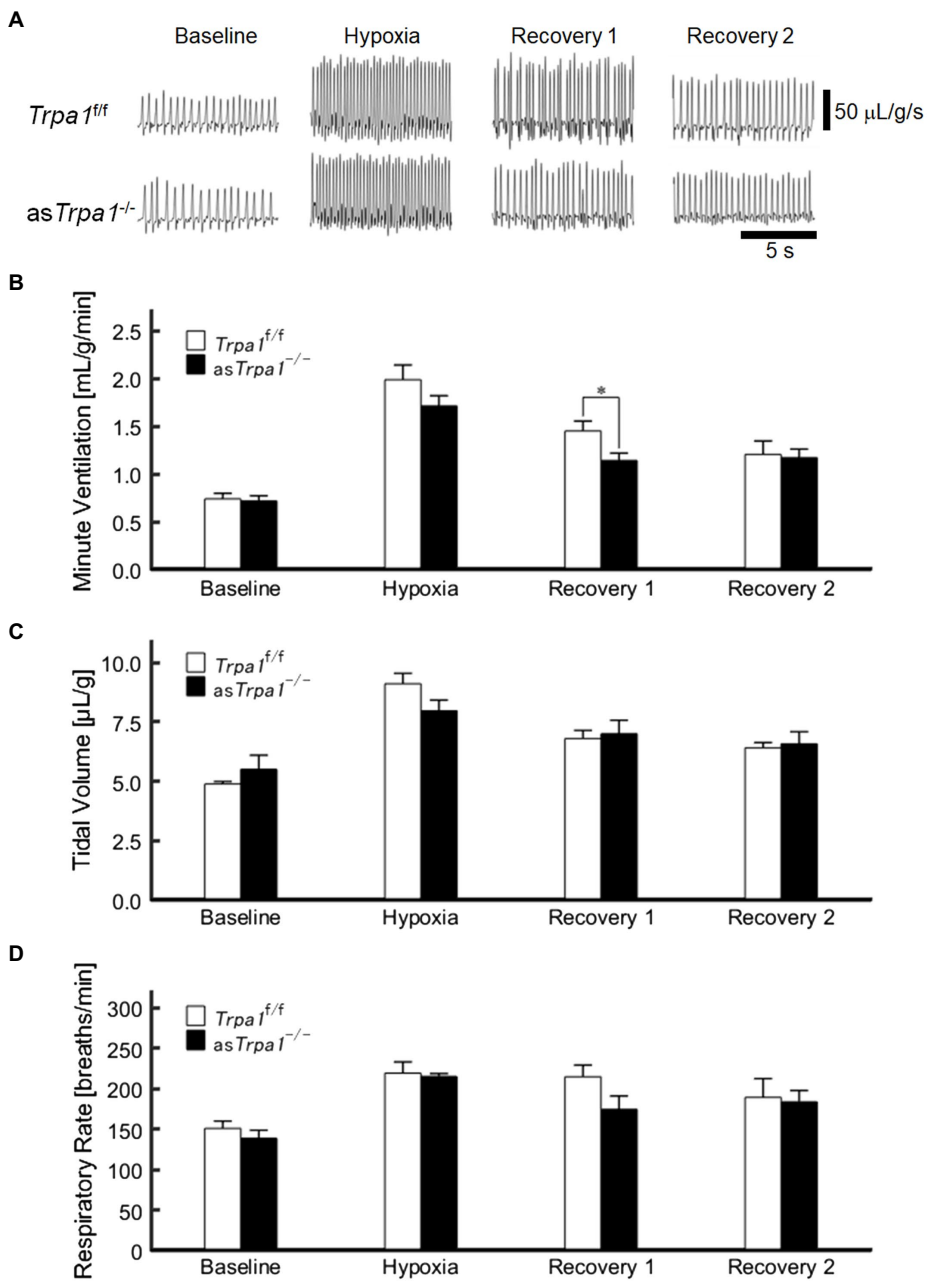
Hypoxic ventilatory responses in as*Trpa1*^−/−^ and *Trpa1*^f/f^ mice. **(A)** Representative recordings of respiratory flow (inspiration upward) in room air (baseline), 7% hypoxia, Recovery 1 (first 5min), and Recovery 2 (second 5min). In both as*Trpa1*^−/−^ and *Trpa1*^f/f^ mice, minute ventilation (V_E_) increased during hypoxia and then decreased in Recovery 1, remaining significantly elevated over the baseline level throughout both recovery phases. In Recovery 1, V_E_ was smaller in as*Trpa1*^−/−^ than *Trpa1*^f/f^ mice **(B)** Minute ventilation (V_E_) in *Trpa1*^f/f^ (*n*=12) and as*Trpa1*^−/−^ (*n*=6) mice in successive ventilatory phases. Of note, V_E_ was significantly smaller in as*Trpa1*^−/−^ than *Trpa1*^f/f^ in Recovery 1 (*p*=0.034). **(C)** Tidal volume (V_T_) in successive ventilatory phases. There was a significant interaction between transient receptor potential ankyrin 1 (TRPA1) conditions (as*Trpa1*^−/−^ and *Trpa1*^f/f^)×ventilatory response phases (*F*_3,48_ in as*Trpa1*^−/−^ and *Trpa1*^f/f^ mice=3.318, *ε*_GG_=0.658, *p*<0.05) but not between TRPA1 condition and V_T_ in any of the ventilatory phases. Pairwise comparisons in as*Trpa1*^−/−^: Baseline vs. Hypoxia (*p*<0.001), Baseline vs. Recovery 1 and Baseline vs. Recovery 2 (both *p*<0.01), and Hypoxia vs. Recovery 2 (*p*<0.05) and in *Trpa1*^f/f^ Baseline vs. Hypoxia (*p*<0.001), Baseline vs. Recovery 1, Baseline vs. Recovery 2, Hypoxia vs. Recovery 1, and Hypoxia vs. Recovery 2 (all *p*<0.01). **(D)** RR in successive ventilatory phases. There was a significant main effect on RR of the ventilatory response phases (*F*_3,48_=17.967, *p*<0.001) but no significant interaction between RR and TRPA1 conditions (*F*_3,48_=1.396, *p*=0.08). There were no significant pairwise differences between ventilatory response phases in either as*Trpa1*^−/−^ or *Trpa1*^f/f^. Data are means±SE. ^*^p<0.05; Bonferroni corrected.

V_E_ profiles in as*Trpa1*^−/−^ and *Trpa1*^f/f^ mice are shown in Figures 4A,B. There was a significant main effect of the ventilatory response phases [*F*(3, 48)=85.011, *p*<0.001] but not between the TRPA1 conditions [*F*(1, 16)=1.843, *p*=0.193]. In both as*Trpa1*^−/−^ and *Trpa1*^f/f^ mice, V_E_ increased during hypoxia when compared to the pre-hypoxic baseline level (*p*<0.001) and then decreased in Recovery 1. However, V_E_ stayed above the baseline level throughout the recovery phases in both as*Trpa1*^−/−^ and *Trpa1*^f/f^ mice. Both V_T_ and RR components drove ventilatory changes throughout the hypoxic course in both as*Trpa1*^−/−^ and *Trpa1*^f/f^ mice (Figures 4C,D).

**Figure 5:**
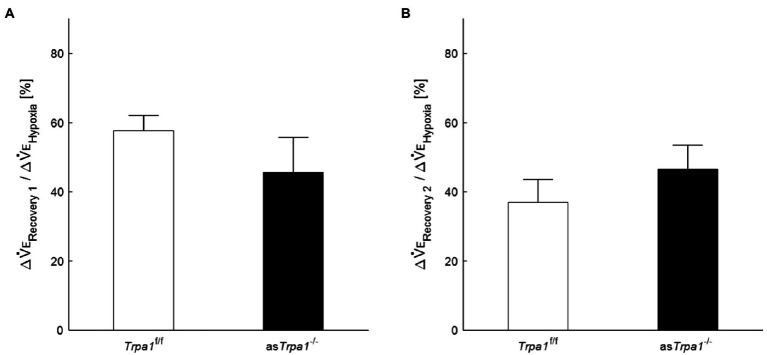
Post-hypoxic persistent respiratory augmentation in astrocyte-specific *Trpa1* knockout mice. The ΔV_ERecovery_/ΔV_EHypoxia_, denoting PHRA, were not significantly different between as*Trpa1*^−/−^ and *Trpa1*^f/f^ in either Recovery 1 **(A)** or Recovery 2 **(B)**. Data are means±SE.

Although there was no significant interaction of TRPA1 conditions×ventilatory phases [*F*(3, 48)=2.352, *p*=0.084], we performed a between-TRPA1 comparison in each phase. V_E_ tended to be smaller in *Trpa1*^f/f^ than as*Trpa1*^−/−^ mice during hypoxia, but the difference was not significant (*p*=0.158). V_E_ became significantly smaller in as*Trpa1*^−/−^ mice during Recovery 1 (*p*=0.034), but the PHRA phenomenon remained noticeable in both as*Trpa1*^−/−^ and *Trpa1*^f/f^ mice (Figure 4B). On average, ΔV_ERecovery_/ΔV_EHypoxia_ percentage values denoting PHRA magnitude were little different between as*Trpa1*^−/−^ and *Trpa1*^f/f^ in post-hypoxic Recovery 1 and 2 phases (Figures 5A,B, respectively). Although we did not conduct statistical analysis because the number of *Trpa1*^f/f^ mice was small, there seems to be a gender difference; ΔV_ERecovery1_/ΔV_EHypoxia_ values in male and female *Trpa1*^f/f^, and male and female as*Trpa1*^−/−^ mice were 52, 61, 67, and 16%, respectively. ΔV_ERecovery2_/ΔV_EHypoxia_ values in these mice were 38, 36, 51, and 40%, respectively.

## Discussion

This study investigated the role of astrocytes in the PHRA, representing short-term potentiation of respiration. The findings show that astrocytes mediate PHRA. Pharmacological blockade of astrocyte activation by AA inhibited PHRA. The knockout as*Trpa*1^−/−^ mice showed less increase in ventilation in response to hypoxia than *Trpa1*^f/f^ mice. However, the magnitude of PHRA was not attenuated in as*Trpa*1^−/−^ when compared to *Trpa1*^f/f^ mice. Our findings demonstrate the putative role of the astrocyte TRPA1 channels in hypoxia sensing, which confirms the recent findings by Uchiyama et al. (2020). We expanded the role of astrocytes to the mediation of PHRA as well. However, TRPA1 detects mild hypoxia (13%) more closely than severe hypoxia (7%; Takahashi et al., 2011; Pokorski et al., 2014). This suggests that PHRA is more likely to occur under conditions of severe hypoxia. Astrocyte-related action on the short-term PHRA occurs through yet unsettled mechanisms other than TRPA1 channels. The involvement of astrocyte TRPA1 channels has been reported in the hippocampal long-term potentiation in mice (Shigetomi et al., 2013). The contribution of these channels may vary depending on the type of brain plasticity.

In the present study, we used AA, as an inhibitory modulator of astrocyte function. AA inhibits the inflammatory response of astrocytes by reducing GFAP and S100 protein synthesis, increasing the expression of the astroglial glutamate transporter GLAST and releases the glutamate receptor antagonist kynurenic acid from astrocytes (Tateishi et al., 2002; Mori et al., 2004; Asano et al., 2005; Wajima et al., 2013; Yamamura et al., 2013; Yanagisawa et al., 2015). We have previously reported that AA delays the occurrence of seizures and prevents respiratory arrest in severe hypoxia (Fukushi et al., 2020).

The present finding of counteracting the PHRA by AA indicates that astrocytes are influential in shaping respiratory neural plasticity. Hypoxia activates the carotid body, and the information is relayed *via* the carotid sinus nerve to the medullary solitary tract nucleus, emanating to other respiratory regions in the brainstem and spinal cord (Guyenet, 2014). Astrocytes around the excited neurons are activated *via* neurotransmitters spilled from neurons. Once activated, they release gliotransmitters that in turn activate respiratory neurons responsible for the sustenance of respiratory potentiation. Of note, the hitherto mechanistic studies on respiratory neural plasticity have been explicitly focused on neurons but not on glial cells. The present study is the first to demonstrate that astrocytes mediate the neural plasticity of respiration.

The short-term potentiation of brain excitability, leading to the continuation of respiratory augmentation after the stimulus cessation, referred to as neural plasticity, has been previously reported (Eldridge, 1973, 1976; Tawadrous and Eldridge, 1974; Eldridge and Gill-Kumar, 1980; Wagner and Eldridge, 1991). The mechanisms of respiratory plasticity are also present in the spinal cord (Feldman et al., 2003; Mitchell and Johnson, 2003; Fuller and Mitchell, 2017). One of the most extensively investigated phenomena in this context is the phrenic long-term facilitation following acute intermittent hypoxia. Regarding the cellular mechanism of facilitation, the Q and S signaling cascades in the phrenic motor nucleus have been proposed, induced by activation of metabotropic receptors coupled to Gq and Gs proteins, respectively, interacting *via* crosstalk inhibition. The serotonin-dependent Q pathway dominates in the phrenic facilitation during mild-to-moderate hypoxia. In contrast, the S pathway is serotonin-independent and dominates during severe hypoxia (Devinney et al., 2013; Fuller and Mitchell, 2017).

Recent studies have revealed an active role of astrocytes in brain plasticity related to other than respiratory functions, with a notable reference to hippocampal memory (Magistretti, 2006; Ota et al., 2013; Croft et al., 2015; Sims et al., 2015; De Pittà et al., 2016). Astrocytes secrete synapse-modulating gliotransmitters such as glutamate, ATP, d-serine, and GABA (Jourdain et al., 2007; Henneberger et al., 2010; Takata et al., 2011; Kang et al., 2013; Shigetomi et al., 2013; Verkhratsky et al., 2016; Zorec et al., 2018; Santello et al., 2019). The regulation of postsynaptic glutamate receptors, particularly α-amino-3-hydroxy-5-methyl-4-isoxazolepropionic acid (AMPA) receptors, is dependent on ATP released from astrocytes. The elevation in astrocytic Ca^2+^, occurring slowly in the order of seconds, stimulates glutamate release which activates astrocytic metabotropic glutamate receptors (Agulhon et al., 2012; Navarrete et al., 2012). The classical form of neural plasticity also depends on *N*-methyl-d-aspartate (NMDA) receptors and Ca^2+^-dependent slow release of d-serine from astrocytes (Henneberger et al., 2010). Further, astrocytes express a variety of receptors such as acetylcholine, ATP, GABA, and endocannabinoids (Porter and McCarthy, 1997; Haydon, 2001; Charles et al., 2003).

There are an increasing number of studies referring to the functional role of astrocytes in respiratory control other than respiratory plasticity. Astrocytes in the brainstem are sensitive to hypoxia and involved in HVR (Tadmouri et al., 2014; Angelova et al., 2015; Marina et al., 2015; Fukushi et al., 2016; Pokorski et al., 2016; Rajani et al., 2018; Uchiyama et al., 2020). Astrocytes in the ventral respiratory network, including the pre-Bötzinger complex, release ATP, which increases respiratory activity during hypoxia, putatively counteracting the depressive effects of hypoxia (Gourine et al., 2005; Marina et al., 2016a; Gourine and Funk, 2017; Funk and Gourine, 2018; Rajani et al., 2018). ATP acts *via* P2Y_1_ receptors in the pre-Bötzinger complex to increase the respiratory burst rate with increases in intracellular Ca^2+^ and glutamate release (Lorier et al., 2007; Huxtable et al., 2010). Astrocytes also are strongly involved in the central control of sympathetic activity and cardiovascular function, including systemic hypertension (Marina et al., 2016b), which are enhanced by acute and particularly repeat hypoxia episodes sensed by carotid chemoreceptors (Prabhakar et al., 2015). There is a biological plausibility that medullary astrocytes, respiratory neurons, and peripheral chemosensing intertwine with each other in shaping PHRA. Alternative study designs are needed to further explore this issue.

In the present study, AA failed to affect V_E_, although RR was increased in mice receiving a high dose of AA in room air. This phenomenon suggests that AA can affect breathing, i.e., inhibition of astrocyte activation may alter breathing patterns. In line with this notion, we showed that HVR was attenuated by a high dose of AA. However, AA blunted PHRA much more, suggesting that PHRA is activity-dependent plasticity.

There may be a concern over the time-dependent stability of minute ventilation on the background of a high dose of AA. Our additional investigation revealed that minute ventilation was stable over 240min in this condition (Supplementary Figure 1). Likewise, another set of control investigations showed that hypoxia loadings repeated three times provide close reproducibility (Supplementary Figure 2).

One potential limitation of this study could be a lack of the animal’s temperature control. In the classical “closed chamber” whole-body plethysmography, tidal volume is calculated by measuring the chamber pressure based on the combined gas law stating that the ratio of the product of gas pressure and volume to the absolute gas temperature is equal to a constant (Drorbaugh and Fenn, 1955). The chamber pressure is recorded while the chamber is sealed, and the body temperature weighs in on the result (Mayer et al., 2014; Rourke et al., 2016; Baby et al., 2018). In practice, however, the body temperature changes are so small during the hypoxic challenges of a couple of minutes that they are usually neglected for the sake of simplicity (Onodera et al., 1997). In the present study, we adopted the “open flow” plethysmography in which the chamber gas is continuously suctioned at a constant flow rate during the continuous recording. We calculated the tidal volume by integrating the airflow whose changes are proportional to those in the chamber pressure (Lundblad et al., 2002). In this case, tidal volume is expressed at ambient temperature (25°C), which obviates the need for taking the animal’s body temperature. Another limitation of this study was that we failed to examine metabolic rate in knock-out mice or its potential alterations by AA, which could influence respiration. Metabolic aspects require further exploration using alternative study designs.

The ultimate purpose of this research was to refer to the mechanism of post-hypoxic short-term respiratory plasticity in unanesthetized humans, which is essential to get insights into the pathophysiology of and preventive measures for periodic breathing, e.g., sleep apnea. This purpose stemmed from the studies showing that PHRA is involved in the mitigation of periodic breathing in sleep apnea (Georgopoulus et al., 1992; Mahamed and Mitchell, 2007; Mateika and Syed, 2013; Mateika and Komnenov, 2017) and heart failure (Ahmed et al., 1994); the notion supported in a computer simulation study (Eldridge, 1996). Our results showed that astrocytes, but not the astrocytic TRPA1 channel, were involved in the development of PHRA, suggesting that the TRPA1 is engaged in shaping HVR but not PHRA. The TRPA1 channel likely plays a (patho)physiological role in acute hypoxic conditions such as an attack of bronchial asthma (Shen et al., 2012). In diseases with periodic breathing such as sleep apnea, astrocytes may contribute to its prevention by exerting PHRA. Additionally, the observation that ΔV_ERecovery1_/ΔV_EHypoxia_ tended to be smaller in the female as*Trpa1*^−/−^ mice raises the implication of a greater role of astrocytic TRPA1 in the female gender, which requires further exploration.

In conclusion, we have provided novel aspects of PHRA’s role linking it to astrocyte activation and suggesting that this tandem arrangement contributes to respiratory stability and potentially might be influential in the prevention of periodic breathing. However, caution should be exercised in the translation of animal findings to human settings before further exploratory research. We conclude that astrocytes mediate the post-hypoxic persisting respiratory augmentation by mechanisms other than the hitherto recognized role of TRPA1 channels in hypoxia sensing.

## Data Availability Statement

The original contributions presented in the study are included in the article/Supplementary Material, further inquiries can be directed to the corresponding author.

## Ethics Statement

The animal study was reviewed and approved by Ethics Committee for Animal Experiments of the Murayama Medical Center.

## Author Contributions

IF conceived and designed the study, performed the animal experiments, analyzed the data, and drafted the manuscript. KT performed the statistical analysis and drafted the manuscript. MP edited and revised the manuscript. YK, MY, and YH participated in the design of the study. AN and YM provided the animals and revised the manuscript. HO supervised the experiments and revised the manuscript. YO conceived and designed the study, analyzed the data, and revised the manuscript. All authors contributed to the article and approved the submitted version.

## Funding

This work was supported by JSPS KAKENHI (17K08559, 18K17783, 19K17386, 19K17620, 20K19368, and 20K19474) and the Japanese Physical Therapy Association (JPTA2019 and JPTA2020).

## Conflict of Interest

The authors declare that the research was conducted in the absence of any commercial or financial relationships that could be construed as a potential conflict of interest.

## Publisher’s Note

All claims expressed in this article are solely those of the authors and do not necessarily represent those of their affiliated organizations, or those of the publisher, the editors and the reviewers. Any product that may be evaluated in this article, or claim that may be made by its manufacturer, is not guaranteed or endorsed by the publisher.
