# Supplementary material for: Activation of Astrocytes in the Persistence of Post-hypoxic Respiratory Augmentation
Source: Front Physiol. 2021 Oct 8;12:757731. doi: 10.3389/fphys.2021.757731 (PMC8531090; doi:10.3389/fphys.2021.757731)
Supplement: Supplementary file 1 [file Data_Sheet_1.pdf]

SUPPLEMENTARY FIGURE 1

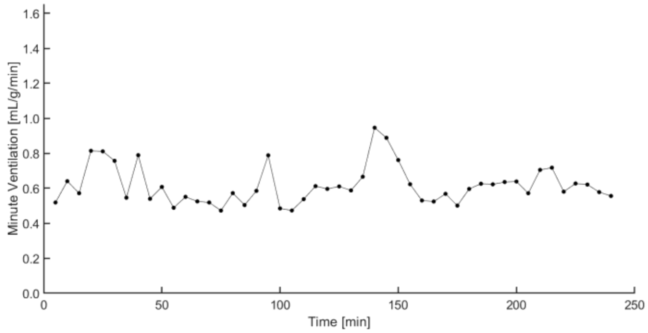

Effects of arundic acid (AA) on ventilation in mice.

We pretreated mice (n=2, male, aged 24 weeks) with AA (300 mg/kg) continuously measuring ventilation in room air by whole-body plethysmography for 240 min. The time-course of minute ventilation was plotted out. Minute ventilation had a stable course after AA.

SUPPLEMENTARY FIGURE 2

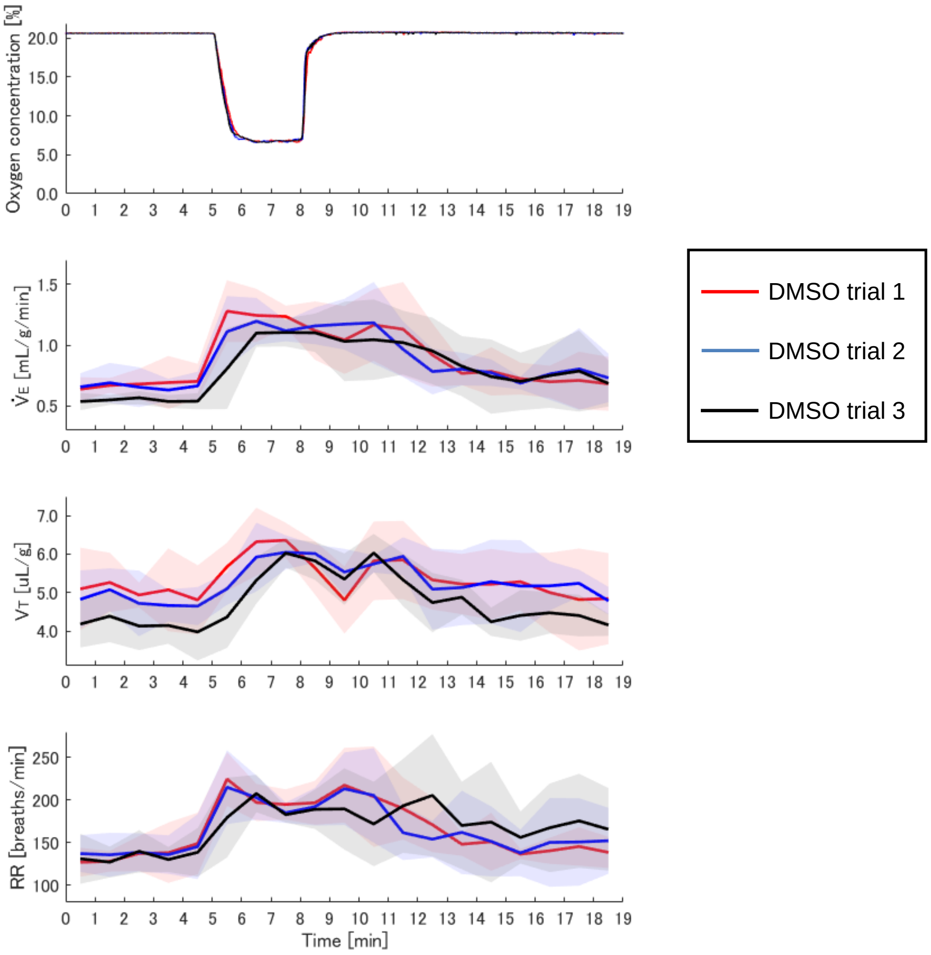

Effects of repeated hypoxic exposure on ventilatory responses.

We examined ventilatory variability in response to hypoxia after dimethyl sulfoxide (DMSO) injections (DMSO trial 1-3: DMSO 0.45 µL/g, 0.45 µL/g, 0.9 µL/g) in mice (n=5, male, aged 34.2 ± 4.2 weeks). DMSO was diluted in saline and given i.p. First, the mouse was acclimatized to the recording chamber for 60 min. Then, after recording the baseline normoxic data for 5 min, the chamber O_2 was rapidly lowered to 7%, which was maintained for 2 min followed by a switchback to room air. The recovery was recorded for 10 min. This protocol was repeated after DMSO injections at 30-min intervals. Traces from top to bottom are oxygen concentration in the chamber, minute ventilation ($\dot{V}_E$), tidal volume (V_T) and respiratory rate (RR). The variables in room air, 7% O_2, or the recovery did not appreciably change over the three DMSO injections. Note that the mice in these experiments were older than those in the main experiments with AA injection. Data are means (± SD).
